# Setting Time and Strength Monitoring of Alkali-Activated Cement Mixtures by Ultrasonic Testing

**DOI:** 10.3390/ma14081889

**Published:** 2021-04-10

**Authors:** Biruk Hailu Tekle, Ludwig Hertwig, Klaus Holschemacher

**Affiliations:** Structural Concrete Institute (IfB), Leipzig University of Applied Sciences (HTWK Leipzig), Karl-Liebknecht-Str. 132, 04277 Leipzig, Germany; birukh.tekle@gmail.com (B.H.T.); ludwig.hertwig@htwk-leipzig.de (L.H.)

**Keywords:** alkali-activated cement, geopolymer, ultrasonic testing, setting time, compressive strength, Taguchi design

## Abstract

Alkali-activated cement (AAC) is a promising binder that replaces ordinary Portland cement (OPC). In this study, the development of setting time and strength of AAC mixes were studied using ultrasonic testing method. The test results were compared with traditional Vicat setting time and compressive and flexural strengths. The findings showed that setting times and strengths have a strong correlation with ultrasonic velocity curve. The initial setting time corresponds well with the ultrasonic velocity curve’s dormant period, and the final setting time with the time it takes to reach the velocity curve’s maximum acceleration. Both setting times also showed a correlation with the value of the maximum acceleration. An exponential relation was found between the ultrasonic velocity and the compressive and flexural strengths. The effect of binder content, alkaline solid to binder ratio (AS/B), sodium silicate to sodium hydroxide solids ratio (SS/SH), and total water to total solid binder ratio (TW/TS) on the strength and setting time are also studied using Taguchi method of experimental design. AS/B ratio showed a significant influence on the setting time of AAC while TW/TS ratio showed only a minor effect. The ultrasonic velocities were able to capture the effect of the different parameters similar to the compressive strength. The velocity decreased mainly with the increase of TW/TS ratio and binder content, while AS/B and SS/SH ratios showed a lower influence.

## 1. Introduction

The need to replace ordinary Portland cement (OPC) is stronger than ever. This is due to the environmental problems associated with the binder. Alkali-activated cement (AAC) is one of the preponderant promising alternative binders to OPC. It is produced by activating source materials such as fly ash and slag with alkaline solutions. Its main advantage is its environmental benefits, with 80% or greater reduction in CO_2_ emission compared to OPC [1,2]. Due to the utilization of mainly by-product materials and zero cement use, AAC has been attracting researchers for decades [3,4].

AAC’s mechanical and durability performances have been reported to be at least on par with OPC [5,6,7]. Despite such promises, the large-scale utilization of AAC concrete still faces challenges because of widely varying source materials [8], lack of standard mix design methods [9], and use of corrosive alkaline solutions [10]. Hence, different aspects of AAC concrete are still open for research.

Most of the studies on AAC are mainly on heat-cured AAC systems and are focused on mechanical performance. Heat curing limits the applicability of AAC. Ambient cured AACs have wider application areas. The use of ground granulated blast furnace slag (GGBS) is common for making AAC cured at ambient conditions [11]. The high calcium oxide content and fineness of GGBS improves the binder system’s reactivity and enhance reaction product formation at ambient temperature. However, the use of GGBS reduces the setting time and workability of the concrete [12,13,14]. This is due to its high calcium content, resulting in calcium aluminosilicate hydrate (C-A-S-H) gel at early duration [13]. Such low setting time and workability limit the application of AAC. Hence, it is essential to produce ambient cured AAC concrete with a better setting time and workability. To achieve this, it is critical first to understand how the various constituents of AAC affect its setting and strength development. The main factors influencing the setting and strength behavior of AAC are binder content, alkali to binder ratio, activating solution’s concentrations and ratios, and water content [13,14,15].

Setting and strength development can be studied by using traditional methods such as the Vicat needle test [16] or compression tests [17]. However, these methods do not provide much detail and do not allow continuous monitoring of the behavior. Ultrasonic wave technology is a non-destructive test method that has been successfully used to monitor the setting process [18,19,20,21], compressive development [22,23], and properties such as internal defect and crack propagation of concrete [24,25]. These measurements can be made continuously, providing more detailed insights into the behavior. Ultrasonic wave is sent through a mixture, and after traveling through, a signal is received by the ultrasonic receiver. The strength of the wave received changes with the hardening process of the mixture. Cao et al. [26] reported the particular suitability of ultrasonic testing for AAC systems considering their quick setting and rapid hardening characteristics. Despite such potential, however, only a few studies have been carried out on AACs.

Ultrasonic wave velocity is sensitive to the formation of reaction products such as hydrates and polymers; hence, can characterize very early microstructural changes associated with the setting process. The study of AAC using this method can provide valuable information on the binder’s setting and hardening process. As observed in OPC, this testing method can give significantly better results than traditional methods, especially in the field of in-place evaluation of structural concrete. Uppalapati et al. [27] studied the setting process of alkali-activated GGBS and fly ash (FA) blend using ultrasonic testing. They suggested that specific ultrasonic velocity ranges can be used to estimate the Vicat initial and final setting times. Chen et al. [28] investigated the setting and nanostructural evolution of metakaolin-based AAC using ultrasonic wave reflection tests. Suraneni et al. [29] investigated AAC’s setting time using penetration resistance and ultrasonic shear wave reflection. Their results revealed that AAC has a wide range of setting times depending on source materials and chemical parameters such as silica to alumina and water to alkali ratios. To fully characterize the setting process of AAC, they recommended using both test methods. Buchwald et al. [30] investigated the reaction progress of metakaolin-GGBS blends activated by sodium hydroxide using ultrasonic velocity. The ultrasonic measurement was able to illustrate the early phase formations very well.

This study extends the use of ultrasonic testing into the field of blended binder system AAC. The ultrasonic velocity method is used to investigate the setting time of FA-GGBS-silica fume (SF) mixes activated by sodium silicate and sodium hydroxide. The evolution of compressive and flexural strengths with the ultrasonic velocity, which is little studied for AAC, is investigated in detail. Furthermore, a parametric study was conducted using both traditional testing methods and the ultrasonic method to understand the effect of mix proportion parameters and investigate the ultrasonic method’s suitability for parametric study. The effect of binder content, alkaline solid to binder ratio (AS/B), sodium silicate to sodium hydroxide solids ratio (SS/SH), and total water to total solid binder ratio (TW/TS) on the strength and setting time are studied.

## 2. Experimental Program

### 2.1. Materials

FA, GGBS, and SF were used as the source materials. All the source materials are commercially available and comply with the EN 450-1 [31], EN 15167-1 [32], and EN 13263-1 [33] requirements. Table 1 summarizes the chemical compositions of the source materials. The chemical analysis was performed using Energy Dispersive X-Ray analysis (EDX) (Noran System SIX, Berlin, Germany) with a scanning electron microscope (Jeol JSM–IT 100, JEOL Ltd., Freising, Germany). The activator solution used is a mixture of sodium silicate and sodium hydroxide. The sodium silicate solution includes 26.82% silicate, 8.2% sodium oxide, and 64.98% water. The sodium hydroxide is a 50% by weight solution. A fine aggregate with a maximum aggregate size of 2 mm was used. 

### 2.2. Experimental Plan and Mix Proportion

Table 2 shows the mixes used for the experimental program. The mixes were designed based on the Taguchi method of experimental design. This is a fractional factorial design method that uses a special set of orthogonal arrays (OA) to design experiments. Due to the OA, the method provides significantly reduced variance for the experiment making it possible to investigate many variables with a small number of experiments. The design of experiments using OA is quite efficient compared to traditional experiment designs [34,35]. The method has been successfully used in concrete technology for the investigation of the physical and mechanical properties of concrete [34,35,36,37,38,39]. In this study, it is used to investigate the influence of mix design parameters on the ultrasonic velocity, setting time, and compressive and flexural strengths of AAC mixtures. 

Four parameters, binder content (B), the alkaline solid to binder (AS/B) ratio, sodium silicate to sodium hydroxide solids (SS/SH) ratio, and total water to total solid (TW/TS) ratio, were considered for the analysis. Alkaline solid (AS) is the solid portion of the alkaline solutions; only this portion is varied to avoid the effect of water, which happens when the alkaline liquid to binder ratio is varied. TW is the sum of water from the alkaline solution and the free water. TS is the total amount of solid binders in the mixture, i.e., AS and the source materials. Each of the parameters was varied in three levels. Binder content of 550 kg/m^3^, 650 kg/m^3^ and 750 kg/m^3^, AS/B ratio of 0.14, 0.18, and 0.22, SS/SH ratio of 1.5, 2.0, and 2.5 and TW/TS ratio of 0.29, 0.34, and 0.39 were used. Hence, the Taguchi three level four factor design was selected to design the experiment. The most appropriate orthogonal array for this is L9 array as per the Taguchi method [40], which has a total of 9 runs. Hence, nine Taguchi mixes (TM) were designed. The mix proportion of each of the Taguchi mix is as shown in Table 2. This table also shows the alkaline solution, water, and sand required to make the specific TM mix. For practical reasons, alkaline solutions are used instead of solids when preparing the mixture. However, the amount of solid (instead of solution) is taken as a parameter for analysis. This is done so to avoid the effect of water, which happens when varying solution ratios. The binder used is 55% FA, 40% GGBS, and 5% SF. This proportion is based on preliminary mixes. As per the chemical composition in Table 1, this proportion of source materials results in a CaO content of about 20%. Mixes with such binder result in aluminum modified calcium silicate hydrate (C-A-S-H) along with aluminosilicate polymers phases; sodium aluminosilicate hydrate (N-A-S-H), leading to a suitable coexistence of N-A-S-H and C-A-S-H [41]. According to Herrmann et al. [41], this results in a good compromise between strength and durability.

### 2.3. Specimen Preparation and Test Methods

At least 24 h before mixing, sodium silicate and sodium hydroxide solutions were mixed in the required proportion. The specimens were prepared by mixing the dry materials (sand and binder) in a mixer for about two minutes. The alkaline solution was then mixed with the additional water and added slowly to the dry mixture and mixed for about four minutes.

Each of the nine Taguchi AAC mixes was tested using an ultrasonic testing method according to EN 12504-4 [42]. The measurement was performed using IP-8 ultrasonic measuring instrument (UltraTest). The instrument consists of a PC-connected controller unit allowing up to eight measuring cell connections. Molds with a volume of 95 cm^3^ were used. An ultrasonic wave was transmitted every minute to study the setting and strength development of the mixes. By measuring the time it takes from the transmitter to the receiver and the length between the two, the ultrasonic velocity (length/time) was computed. Measurements were conducted for at least two days for each of the mixes.

Compressive and flexural tests were performed on 40 mm × 40 mm × 160 mm prism samples according to EN 196-1 [17]. The tests were carried out at 10, 24, 35, and 48 h after casting. The setting times were evaluated using the Vicat needle apparatus. The initial setting time was taken as the elapsed time between mixing the solution and binder system to the time at which the distance between the needle and baseplate is about 3 mm (37 mm penetration depth), and the final setting time between the mixing to the time at which the needle penetrates less than 0.5 mm into the specimen. The setting time measurements were conducted at a similar temperature setting as the ultrasonic tests, an average temperature of 23 °C.

## 3. Results and Discussions

### 3.1. Setting Process

#### 3.1.1. Vicat Setting Time

**Figure 1:**
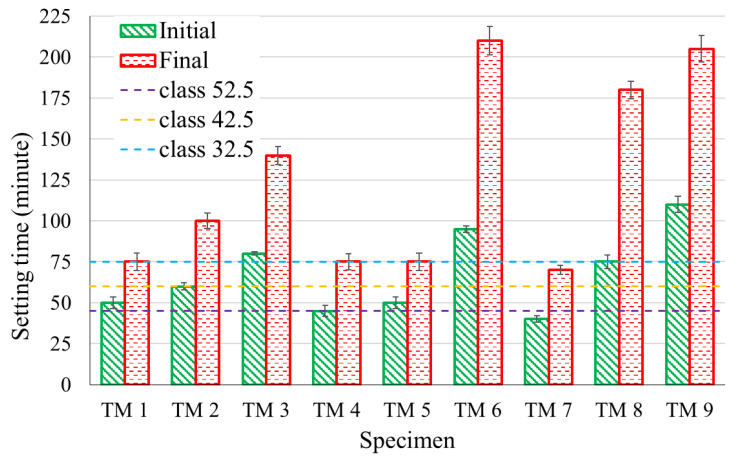
Initial and final setting times according to Vicat setting time test.

Figure 1 shows the mean and standard deviation (SD) of the initial and final setting time according to the Vicat method for each of the studied mixes. As per EN 197-1 [43], a standard cement’s initial setting time may not be less than 45, 60, and 75 min for strength class 52.5, 42.5, and 32.5 cement, respectively. The values in Figure 1 show that some of the mixes do not meet this requirement. This is a common problem in AAC with GGBS, and researches are underway to develop a suitable retarder [44,45].

**Figure 2:**
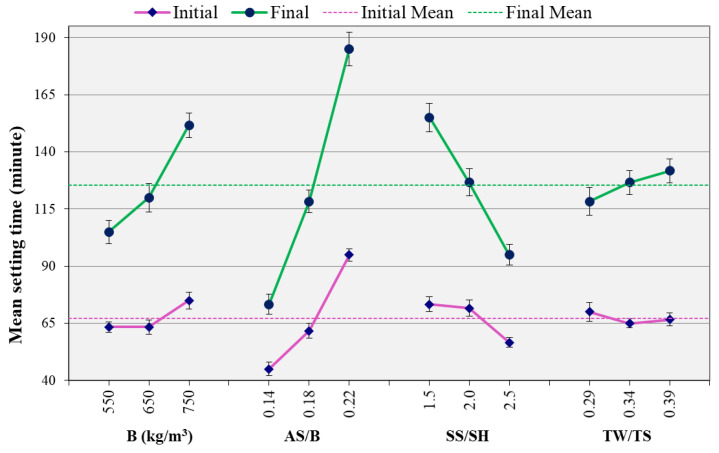
The effect of parameters on the setting time.

Figure 2 shows the effect of each of the studied parameters on the initial and final setting times. Each parameter in this figure is obtained by taking the average of the setting times for all the mixes containing that parameter. For instance, for binder content of 550 kg/m^3^, a final setting time of 75, 100, and 140 min were measured for TM 1, TM 2, and TM 3, as shown in Figure 1. Hence the mean setting time for this parameter is 105 min, as shown in Figure 2.

The setting time showed a considerable variation between the mixes. This is because of the different parameters of the mixes. As shown in Figure 2, the main parameter that significantly affected the setting time is the AS/B ratio. The increase of this ratio increased both initial and final setting times. The binder content and the SS/SH ratio also affected the setting time, however, at a lower significance. An increase of binder content increased the setting time while the reverse was observed for SS/SH. However, unlike OPC mixes where setting time increases with water to cement ratio [22,46,47], water (TW/TS) does not significantly influence the setting time of AAC mixes.

#### 3.1.2. Evolution of Ultrasonic Velocity with Time

**Figure 3:**
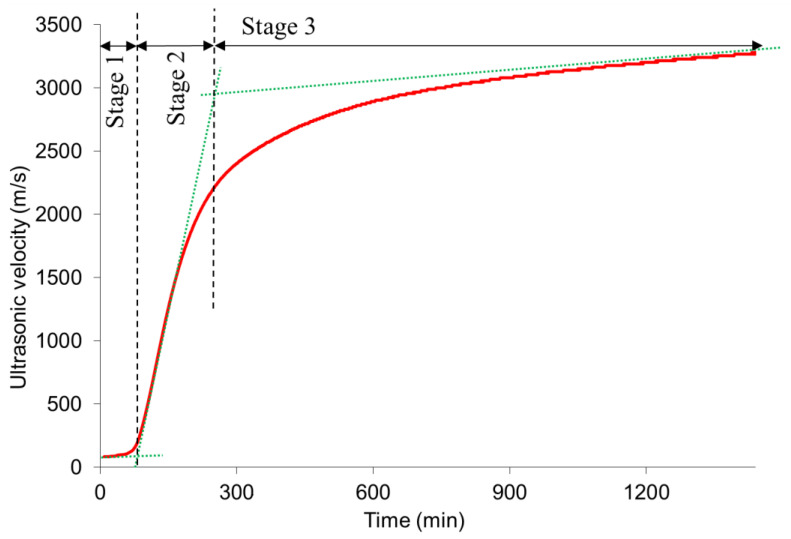
Typical ultrasonic velocity curve (TM 9).

Figure 3 shows the typical evolution of the ultrasonic velocity curve during the first 24 h. The curve can be divided into three main stages [26,48,49]. The first stage is the dormant period. A constant low velocity, an average of about 90 m/s, is observed initially during this stage. The low velocity is due to the higher amount of air bubbles present in the fresh mixture making it difficult for the ultrasonic wave to propagate through the water like viscous suspension [19,50,51]. At this stage, the velocity is lower than the alkali solution’s ultrasonic wave velocity, which is about 1450–1600 m/s [27] and that of air (340 m/s) [18]. During this stage, the ultrasonic waves have difficulties propagating through the liquid viscous suspension because of strong reflections and attenuation from the air entrapped in the mixture [19,48,52]. The presence of air bubbles due to the mixing solution or tiny bubbles entrapped in the paste during mixing is suggested to cause a lower value of ultrasonic velocity [18,53]. This, according to Rapoport et al. [21], can also be explained by the highly elongated length of the wave-path because of the suspended solid grains in the fresh material. The dormant stage corresponds to the wetting and dissolution of the source materials, hydrolysis of silicate and aluminate oligomers or monomers in the solution, and their complexation with calcium or sodium [27,54].

After the dormant stage, the velocity increases rapidly, second stage or acceleration stage, and then gradually (third stage), forming an S-shaped curve. The rapid increase in the second stage is due to the formation and connection of new reaction products. It is well established that AAC’s setting and hardening occur due to condensation between aluminate and silicate species [54]. Due to the condensation, the mixture changes from liquid suspension to solid networks. The ultrasonic wave starts propagating through the connected solid volume, resulting in a higher ultrasonic velocity [55]. In the third stage, the velocity increases slowly and tends to level gradually. The still increasing velocity is due to filling the capillary pores in the compact matrix with further reaction products [26,51].

#### 3.1.3. Vicat Setting Time and Ultrasonic Measurement Comparison

Different methods have been proposed to relate the Vicat’s initial and final setting times to the ultrasonic curve [19,20,51,55]. One such method is merely defining threshold values of ultrasonic velocity for both initial and final setting times. Different construction materials are reported to have significantly different threshold values depending on their composition and the test equipment and excitation frequencies used. Threshold values in the range of 800 to 2700 m/s for initial setting time and 1200 to 3180 m/s for final setting time have been suggested [18,19,20]. The overlap between the initial and final setting time and the wide ranges indicates that it is difficult to use specific ultrasonic values to estimate setting times [19]. The other method consists of identifying the characteristic points or inflection points in the ultrasonic curve. Initial setting time has been correlated with the time when the velocity starts increasing, i.e., the start of stage 2 in Figure 3 [19,55,56]. The final setting time has been associated with the maximum acceleration point [49], and also with the beginning of the third stage or the point where the velocity starts to increase more gradually [19]. However, Robeyst et al. [48] reported that the relation between the ultrasonic velocity and the final setting time is less clear.

**Figure 4:**
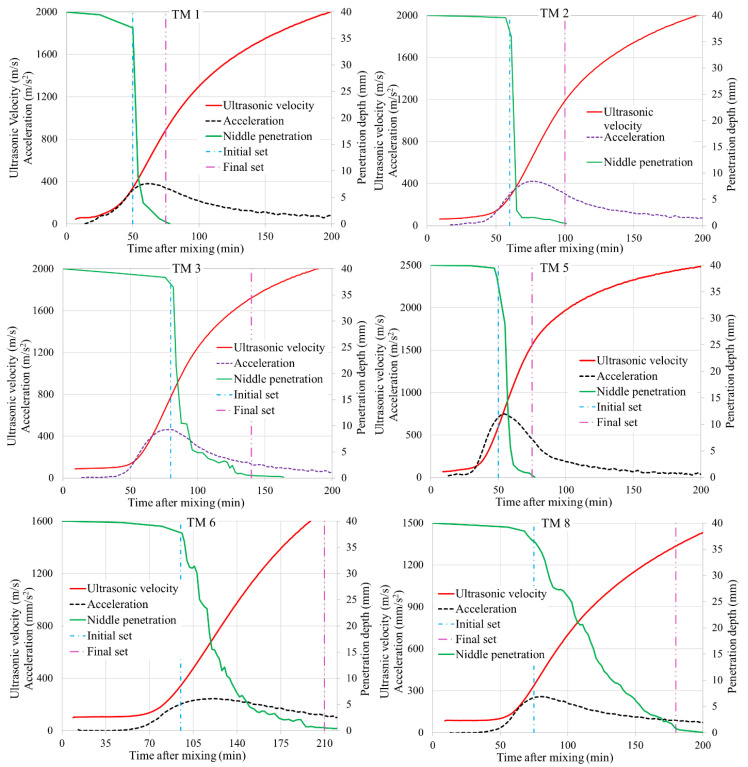
Correction of the ultrasonic curve with penetration depth.

Figure 4 shows the ultrasonic velocity, its derivative (acceleration), and Vicat penetration depth curves for some of the mixes studied. The derivative at a point is calculated by taking the average of four slopes at the point’s immediate vicinity. The initial and final setting points, i.e., the penetration depth of 37 mm and 0.5 mm, are also shown in this figure. In all the mixes except TM 3, the Vicat initial setting time was between the end of stage 1 and the maximum acceleration point of the ultrasonic curve. As shown in Figure 4, in TM 3, the Vicat initial setting time coincides well with the maximum acceleration point. For some of the mixes such as TM 2, the end of stage 1 has correlated well with the initial setting time. During the dormant stage of the ultrasonic curve, the mixes have nearly zero penetration resistance.

**Figure 5:**
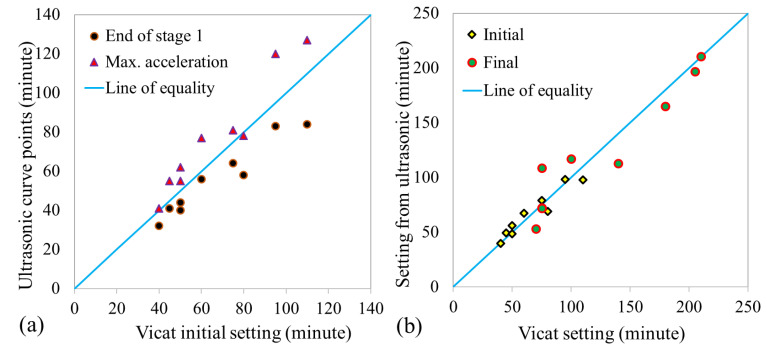
Correlation of setting time measurements (**a**) Vicat initial setting time and ultrasonic curve points, (**b**) Setting times from Vicat and equations.

Figure 5a shows the correlation between the Vicat initial setting time and the end of stage 1 and the maximum acceleration points on the ultrasonic curve. On average, the end of stage 1 on the ultrasonic curve underestimated the initial setting time by about 17%, while the maximum acceleration point overestimated it by the same amount, meaning that the initial setting time is in between these points in the ultrasonic curve.

The final setting time usually corresponds to the maximum acceleration point in the ultrasonic curve [51,55]. However, the current study showed that the maximum acceleration points highly underestimate AAC mixtures’ final setting time. Furthermore, the ultrasonic velocity at the final Vicat setting time varied from 500 to 2000 m/s, showing that the alternative approach of setting a constant ultrasonic velocity to estimate this setting time does not work.

Further analysis of the results showed that the maximum acceleration value varied with both the initial and final setting times. Mixes with high setting time such as TM 6, TM 8, and TM 9 (Figure 1) showed a low maximum acceleration, while those with low setting time such as TM 5 and TM 7 showed high maximum acceleration. This suggests a possible correlation between maximum acceleration and setting time. Using least square optimization, an equation for final setting time based on the maximum acceleration value and the time to reach this acceleration is formulated. Similarly, using the maximum acceleration and the time to reach the end of stage 1, an equation for initial setting time is formulated. These equations are as shown in Equations (1) and (2).

(1) $$t_{f}=11*a_{m}{}^{-1.5}+t_{a}$$

(2) $$t_{i}=7*a_{m}{}^{-0.5}+t_{1}$$

where $t_{f}$ is Vicat final setting time, $t_{i}$ is Vicat initial setting time, $a_{m}$ is maximum acceleration, $t_{a}$ is time at $a_{m}$ and $t_{1}$ is the time at the end of stage 1. Time is in minute, and acceleration is in m/s^2^.

The predicted initial and final setting times using these equations are shown in Figure 5b. A strong coefficient of determination, R^2^ = 0.92 for initial setting time and 0.90 for final setting time, was obtained. This shows the evident relationship between setting time and the ultrasonic curve points, $a_{m}$, $t_{a}$, and $t_{1}$.

### 3.2. Ultrasonic Velocity and Strength Developments

**Figure 6:**
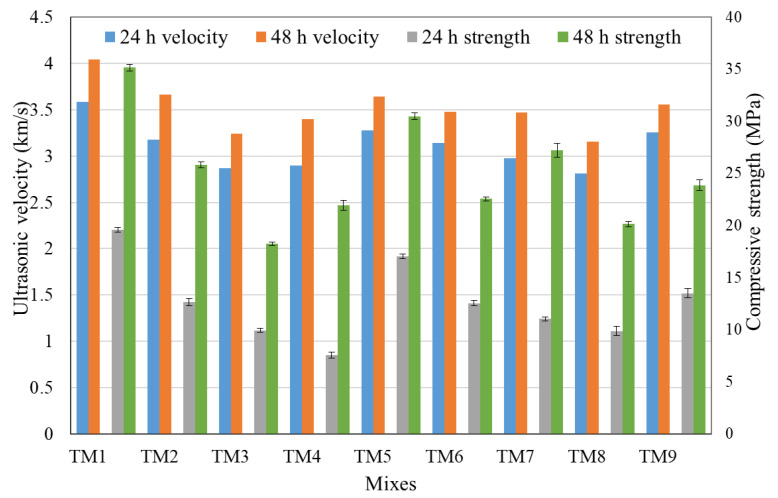
Compressive strength and ultrasonic velocities at 24 and 48 h.

Table 3 shows the ultrasonic velocity and mean compressive and flexural strengths at different ages. The compressive strengths and ultrasonic velocities at 24 and 48 h are also shown in Figure 6. The strengths, as in the case of setting time, showed a considerable variation between the mixes. This is because of the different parameters of the mixes.

**Figure 7:**
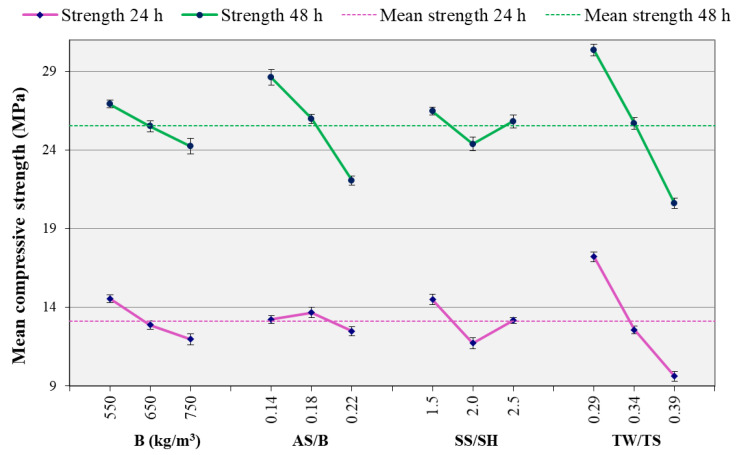
Effect of parameters on compressive strength.

Figure 7 shows the effect of each of the studied parameters on the compressive strength. Each of the figure’s data points is obtained by taking the average of the compressive strengths for all the mixes containing that parameter as explained in Section 3.1.1. As shown in this figure, the main parameter that significantly affects the compressive strength is the TW/TS ratio. The increase of this ratio decreased both the 24 h and 48 h compressive strengths. The relationship is similar to that of OPC concrete’s strength to water to cement ratio relationship. Similarly, the binder content also affected the compressive strength, however, at a lower significance compared to TW/TS ratio. As for the AS/B ratio, the strength decreased significantly in the case of 48 h, but no significant change was observed at 24 h.

**Figure 8:**
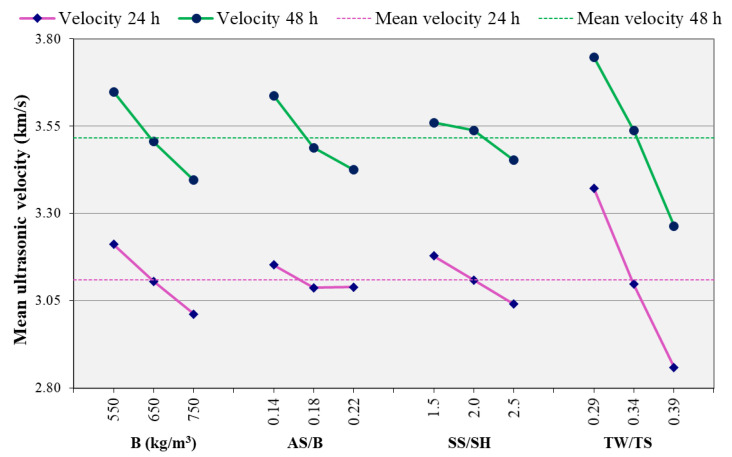
Effect of parameters on ultrasonic velocity.

The influence of each of the parameters on the ultrasonic velocities at 24 h and 48 h is also studied. The results are as shown in Figure 8. Similar trends with the compressive strength were observed. The effect of TW/TS is much more pronounced than the other parameters. From the ultrasonic point of view, high water to binder ratio reduces ultrasonic velocity [52]. This results in a lower ultrasonic velocity of mixes with high TW/TS ratios, as observed in Figure 8.

**Figure 9:**
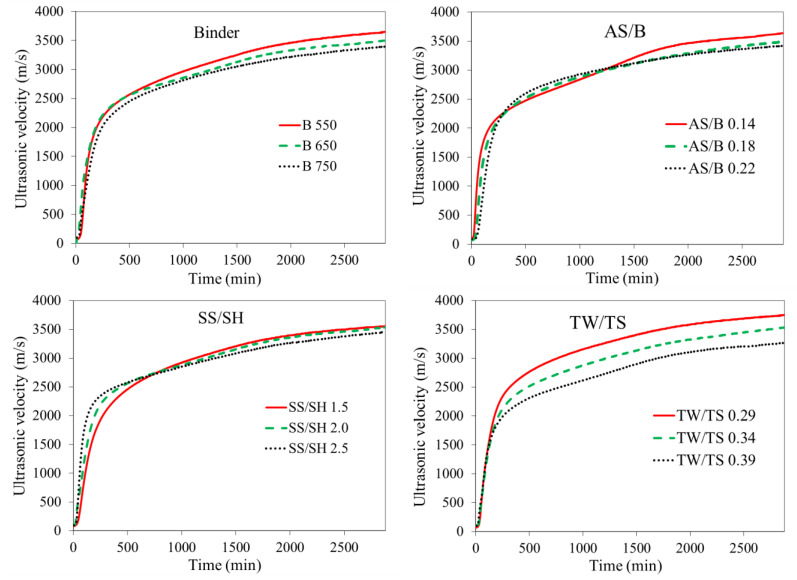
Effect of parameters on ultrasonic velocity.

Figure 9 shows the average ultrasonic velocities for each of the parameters. Each curve is determined by averaging the ultrasonic curves for that parameter. For instance, for the TW/TS ratio of 0.39, three curves, i.e., TM 3, TM 4, and TM 8 (can be referred from Table 2), are available; hence, the curve for TW/TS 0.39 is obtained by averaging these curves. As can be observed from this figure, the TW/TS ratio showed a distinct variation between the three levels for most of the curve, showing the strong influence of this ratio. This graph shows the potential of using the ultrasonic velocity curve for continuous analysis of parameters. Only limited ages can be studied in the case of traditional strength test methods.

As shown in Figure 7 and Figure 9, higher binder content resulted in lower compressive strength. At the investigated ages, the binder’s reaction with the alkaline solution is still at its initial stage. This results in more unreacted binders hence a lower strength as the binder content increased. Furthermore, as can be observed from Table 2 to keep unit volume, mixes with lower binder content had higher sand content. Higher sand content reduces the effective compressibility (more solid particle), resulting in higher ultrasonic velocity [52,57]. This, in addition to the effect of the unreacted binder, is the reason for the higher ultrasonic velocity of mixes with lower binder content. Thus, as can be observed from Figure 7 and Figure 8, the binder effect is stronger in the case of ultrasonic velocity than compressive strength.

Previous studies reported that the increase in alkaline liquid to binder ratio decreases the concrete’s strength [9,35]. In the current study, it was observed that the increase in AS/B ratio has a similar effect on compressive strength, as can be observed in Figure 7. However, it should be noted that this ratio may also show an increasing pattern. This depends on factors such as the value of the ratio and source material [58]. A similar decreasing pattern was observed for the ultrasonic velocity as the AS/B ratio increased, as shown in Figure 8. In both compressive strength and ultrasonic cases, the AS/B ratio showed different significance levels at the two ages with more significance at 48 h. The SS/SH ratio showed a decreasing trend at both 24 and 48 h but at a lower significance compared to the other parameters. The general similarities observed in the compressive strength and ultrasonic velocity analyses show the possibility of using the ultrasonic velocity for parametric study.

### 3.3. Relationship between Ultrasonic Velocity and Strength

**Figure 10:**
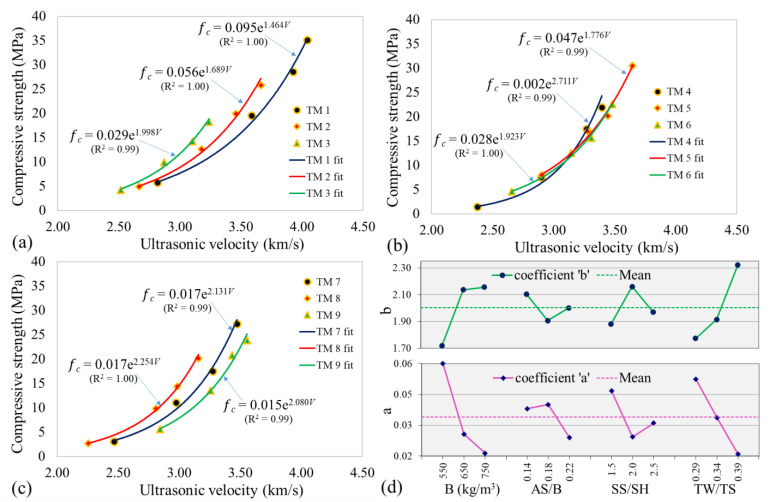
Ultrasonic velocity and compressive strength (**a**) TM 1–TM 3, (**b**) TM 4–TM 6, (**c**) TM 7–TM 9, (**d**) exponential function coefficients.

The relationship between ultrasonic velocity and compressive strength has been defined by either exponential function [22,51,59] or linear function [51,60]. For the early-age behavior of AAC in the current study, an exponential function fits better as shown in Figure 10a–c with a coefficient of determination (R^2^) of 0.99 or 1.00. This means almost all the variation in compressive strength is accounted for by exponential relationship with ultrasonic velocity. The general exponential function is given by:

(3) $$f_{c}=a\cdot e^{bV}$$

where $f_{c}$ is the compressive strength (MPa), V is the ultrasonic velocity (km/s), and a and b are curve fitting coefficients. When a single exponential function was applied for all the specimens in this study (TM 1–TM 9), a lower nevertheless good coefficient of determination (R^2^ = 0.88) with a and b equal to 0.061 and 1.685, respectively, was obtained. The lower value of R^2^ shows that a more accurate correlation between ultrasonic velocity and compressive strength is obtained when each mix is calibrated.

The exponential relation shows that the development of the compressive strength initially falls behind that of the ultrasonic velocity. Meaning the ultrasonic velocity increases while the compressive strength shows no significant change. The increase in ultrasonic velocity is related to condensation between aluminate and silicate species forming large aluminosilicate structural units near the source materials. This results in the change of wave propagation medium. The wave starts to propagate through the solid instead of the liquid [27]. However, the aluminosilicate products generated at this time are limited; hence, no evident strength gain can be obtained.

Figure 10d shows the effect of each of the studied parameters on the curve fitting coefficients a and b. The points for each parameter in this figure are obtained by taking the average of the coefficients for all the mixes containing that parameter similar to the setting times in Section 3.1.1. Binder content and TW/TS ratio showed a strong effect on the coefficients of the exponential function. Coefficient a, similar to compressive strength and ultrasonic velocity, generally showed decreasing pattern as each parameter increases. Coefficient b showed no obvious trend with AS/B and SS/SH ratios. However, for binder content and TW/TS ratio, it showed an increasing trend with each factor, opposite to coefficient a. Coefficient a controls the scale of the exponential function and is the compressive strength value at zero ultrasonic velocity, while b controls the rate of strength increase with respect to ultrasonic velocity. A higher value of b in mixes with lower compressive strength such as those with binder content of 750 kg/m^3^ and TW/TS ratio of 0.39, means that despite their lower strength, they have a higher rate of strength development with respect to the ultrasonic velocity. Coefficient b increased with binder content probably because of the more reacted products with higher binder as the reaction progresses. Higher TW/TS decreases compressive strength and ultrasonic velocity; however, this parameter’s higher value may expedite the reaction rate between the activator and the source material, resulting in a higher b value. An increase in binder content (decrease in sand) decreased the compressive strength, Figure 7. The lower compressive strength could be the reason for the lower value of coefficient a at higher binder content.

**Figure 11:**
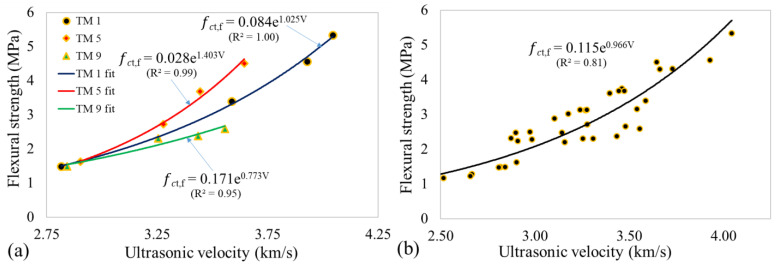
Ultrasonic velocity and flexural strength (**a**) TM 1, TM 5, and TM 9, (**b**) all specimens.

Figure 11a shows the relationship between ultrasonic velocity and flexural strength for the individual mixes. Similar to compressive strength, an exponential relation was observed. A lower yet good correlation (R^2^ = 0.81) was observed when the exponential function was applied for all the flexural results, as shown in Figure 11b. This is, however, lower than the compressive strength value (R^2^ = 0.88). This could be due to the higher sensitivity of flexural tests resulting in higher variability of flexural strength results [61], which lowers the correlation with ultrasonic velocity.

The ultrasonic velocity and its relationship with strength are influenced by the type of source material, aggregate size, type and proportion, the mixture’s air content, curing temperature, and presence of admixtures [19,52,57,62,63]. This study investigated only one type of AAC, and hence the results are limited to the studied AAC parameters. Therefore, it is necessary to accumulate more experimental data considering the various influence factors to improve its reliability.

## 4. Conclusions

Based on the experimental results presented in this study, the following conclusions can be drawn:

- The setting and hardening process of the AAC mixture can be identified into three stages in the ultrasonic curve: the dormant stage, the acceleration stage, and the slow increase stage.
- The main significant parameter affecting the setting time of AAC is the AS/B ratio. Compared to the other factors, TW/TS ratio has only a minor effect on initial and final setting times.
- A good relationship exists between setting time measured by the Vicat needle test and the ultrasonic curves. Initial and final setting times correspond well with the dormant period’s duration and time to reach maximum acceleration, respectively. Both setting times also showed a strong correlation with the maximum acceleration value.
- Exponential relationships were observed between ultrasonic velocity and compressive strength. The relationship has a coefficient of determination (R^2^) of 0.99 when calibrated for individual mixes. This shows that almost all the compressive strength variation is accounted for by an exponential relationship with ultrasonic velocity.
- The ultrasonic velocities were able to capture the effect of different parameters similar to the compressive strength. The velocity decreased mainly with the increase of TW/TS and binder content, while AS/B and SS/SH showed a lower influence.

## Figures and Tables

**Table 1:** Chemical composition of ingredients.

Composition	FA (%)	GGBS (%)	SF * (%)
SiO_2_	49.79	34.48	93.81
Al_2_O_3_	26.71	11.48	0.48
Fe_2_O_3_	8.57	-	1.49
MgO	2.47	7.08	0.46
CaO	4.34	42.43	0.30
K_2_O	3.36	0.66	0.77
Na_2_O	1.28	0.56	0.42
SO_3_	1.49	2.17	0.20
TiO_2_	1.23	1.14	-

* manufacturer specification.

**Table 2:** Mix proportions for each Taguchi mix.

Mix	TM1	TM2	TM3	TM4	TM5	TM6	TM7	TM8	TM9
B (kg/m^3^)	550	550	550	650	650	650	750	750	750
AS/B	0.14	0.18	0.22	0.14	0.18	0.22	0.14	0.18	0.22
SS/SH	1.50	2.00	2.50	2.00	2.50	1.50	2.50	1.50	2.00
TW/TS	0.29	0.34	0.39	0.39	0.29	0.34	0.34	0.39	0.29
SSL (kg/m^3^)	132	189	247	173	239	245	214	231	314
SHL (kg/m^3^)	62	66	69	61	67	114	60	108	110
Water (kg/m^3^)	63	65	67	147	31	53	121	142	2
Sand (kg/m^3^)	1453	1331	1208	1053	1218	1067	938	769	970

B: binder with 55% FA, 40% GGBS, and 5% SF. SHL and SSL are solutions of sodium hydroxide and sodium silicate.

**Table 3:** Ultrasonic velocity and compressive and flexural strength results.

Mix	Ultrasonic Velocity (km/s)				Compressive Strength (MPa)				Flexural Strength (MPa)			
	10 h	24 h	35 h	48 h	10 h	24 h	35 h	48 h	10 h	24 h	35 h	48 h
TM 1	2.82	3.59	3.93	4.04	5.72	19.58	28.56	35.15	1.49	3.32	4.56	5.34
TM 2	2.68	3.19	3.46	3.66	4.89	12.62	19.91	25.82	1.29	3.02	3.75	4.30
TM 3	2.52	2.89	3.10	3.24	4.25	9.94	14.20	18.24	1.17	2.32	2.89	3.13
TM 4	2.37	2.90	3.27	3.39	1.40	7.55	17.45	21.94	-	2.48	3.12	3.61
TM 5	2.89	3.26	3.44	3.65	7.97	17.03	20.16	30.49	1.63	2.72	3.68	4.51
TM 6	2.67	3.13	3.31	3.46	4.59	12.54	15.65	22.56	1.23	2.48	2.31	2.66
TM 7	2.50	3.01	3.27	3.47	3.10	11.04	17.56	27.20	-	2.50	3.13	3.68
TM 8	2.28	2.81	2.98	3.16	2.68	9.87	14.40	20.14	-	1.48	2.28	2.21
TM 9	2.90	3.28	3.43	3.56	5.63	13.50	20.74	23.87	1.49	2.31	2.38	2.59

## Data Availability

The data presented in this study are available on request from the corresponding author.

## References

[1] Duxson P., Provis J.L., Lukey G.C., van Deventer J.S.J. (2007). The role of inorganic polymer technology in the development of ‘green concrete’. Cem. Concr. Res..

[2] Juenger M.C.G., Winnefeld F., Provis J.L., Ideker J.H. (2011). Advances in alternative cementitious binders. Cem. Concr. Res..

[3] Pacheco-Torgal F., Castro-Gomes J., Jalali S. (2008). Alkali-activated binders: A review. Constr. Build. Mater..

[4] Provis J.L., Palomo A., Shi C. (2015). Advances in understanding alkali-activated materials. Cem. Concr. Res..

[5] Wang A., Zheng Y., Zhang Z., Liu K., Li Y., Shi L., Sun D. (2020). The Durability of Alkali-Activated Materials in Comparison with Ordinary Portland Cements and Concretes: A Review. Engineering.

[6] Bakharev T., Sanjayan J., Cheng Y.-B. (2003). Resistance of alkali-activated slag concrete to acid attack. Cem. Concr. Res..

[7] Lahoti M., Tan K.H., Yang E.-H. (2019). A critical review of geopolymer properties for structural fire-resistance applications. Constr. Build. Mater..

[8] Xiao R., Jiang X., Zhang M., Polaczyk P., Huang B. (2020). Analytical investigation of phase assemblages of alkali-activated materials in CaO-SiO2-Al2O3 systems: The management of reaction products and designing of precursors. Mater. Des..

[9] Li N., Shi C., Zhang Z., Wang H., Liu Y. (2019). A review on mixture design methods for geopolymer concrete. Compos. Part B Eng..

[10] Luukkonen T., Abdollahnejad Z., Yliniemi J., Kinnunen P., Illikainen M. (2018). One-part alkali-activated materials: A review. Cem. Concr. Res..

[11] Awoyera P., Adesina A. (2019). A critical review on application of alkali activated slag as a sustainable composite binder. Case Stud. Constr. Mater..

[12] Fang G., Ho W.K., Tu W., Zhang M. (2018). Workability and mechanical properties of alkali-activated fly ash-slag concrete cured at ambient temperature. Constr. Build. Mater..

[13] Nath P., Sarker P.K. (2014). Effect of GGBFS on setting, workability and early strength properties of fly ash geopolymer concrete cured in ambient condition. Constr. Build. Mater..

[14] Lee N.K., Lee H.K. (2013). Setting and mechanical properties of alkali-activated fly ash/slag concrete manufactured at room temperature. Constr. Build. Mater..

[15] Nagajothi S., Elavenil S. (2018). Parametric studies on the workability and compressive strength properties of geopolymer concrete. J. Mech. Behav. Mater..

[16] EN 196-3 (2017). Methods of Testing Cement-Part. 3: Determination of Setting Times and Soundness.

[17] EN 196-1 (2016). Methods of Testing Cement-Part. 1: Determination of Strength.

[18] Trtnik G., Turk G., Kavčič F., Bosiljkov V.B. (2008). Possibilities of using the ultrasonic wave transmission method to estimate initial setting time of cement paste. Cem. Concr. Res..

[19] Trtnik G., Gams M. (2014). Recent advances of ultrasonic testing of cement based materials at early ages. Ultrasonics.

[20] Lee H.K., Lee K.M., Kim Y.H., Yim H., Bae D.B. (2004). Ultrasonic in-situ monitoring of setting process of high-performance concrete. Cem. Concr. Res..

[21] Julie R.R., John S.P., Subramaniam V.K., Surendra P.S. (2000). Using Ultrasound to Monitor Stiffening Process of Concrete with Admixtures. ACI Mater. J..

[22] Lee T., Lee J. (2020). Setting time and compressive strength prediction model of concrete by nondestructive ultrasonic pulse velocity testing at early age. Constr. Build. Mater..

[23] Yoon H., Kim Y.J., Kim H.S., Kang J.W., Koh H.-M. (2017). Evaluation of Early-Age Concrete Compressive Strength with Ultrasonic Sensors. Sensors.

[24] Wolf J., Pirskawetz S., Zang A. (2015). Detection of crack propagation in concrete with embedded ultrasonic sensors. Eng. Fract. Mech..

[25] Zhao G., Zhang D., Zhang L., Wang B. (2018). Detection of Defects in Reinforced Concrete Structures Using Ultrasonic Nondestructive Evaluation with Piezoceramic Transducers and the Time Reversal Method. Sensors.

[26] Cao R., Zhang S., Banthia N., Zhang Y., Zhang Z. (2020). Interpreting the early-age reaction process of alkali-activated slag by using combined embedded ultrasonic measurement, thermal analysis, XRD, FTIR and SEM. Compos. Part. B Eng..

[27] Uppalapati S., Vandewalle L., Cizer Ö. (2020). Monitoring the setting process of alkali-activated slag-fly ash cements with ultrasonic P-wave velocity. Constr. Build. Mater..

[28] Chen X., Sutrisno A., Zhu L., Struble L.J. (2017). Setting and nanostructural evolution of metakaolin geopolymer. J. Am. Ceram. Soc..

[29] Suraneni P., Puligilla S., Kim E.H., Chen X., Struble L.J., Mondal P. (2014). Monitoring Setting of Geopolymers. Adv. Civ. Eng. Matls..

[30] Buchwald A., Tatarin R., Stephan D. (2009). Reaction progress of alkaline-activated metakaolin-ground granulated blast furnace slag blends. J. Mater. Sci..

[31] EN 450-1 (2012). Fly Ash for Concrete-Part. 1: Definition, Specifications and Conformity Criteria.

[32] EN 15167-1 (2006). Ground Granulated Blast Furnace Slag for Use in Concrete, Mortar and Grout-Part. 1: Definitions, Specifications and Conformity Criteria.

[33] EN 13263-1 (2005). Silica Fume for Concrete-Part. 1: Definitions, Requirements and Conformity Criteria.

[34] Türkmen İ., Gül R., Çelik C. (2008). A Taguchi approach for investigation of some physical properties of concrete produced from mineral admixtures. Build. Environ..

[35] Hadi M.N.S., Farhan N.A., Sheikh M.N. (2017). Design of geopolymer concrete with GGBFS at ambient curing condition using Taguchi method. Constr. Build. Mater..

[36] Tanyildizi H., Şahin M. (2015). Application of Taguchi method for optimization of concrete strengthened with polymer after high temperature. Constr. Build. Mater..

[37] Teimortashlu E., Dehestani M., Jalal M. (2018). Application of Taguchi method for compressive strength optimization of tertiary blended self-compacting mortar. Constr. Build. Mater..

[38] Mehta A., Siddique R., Singh B.P., Aggoun S., Łagód G., Barnat-Hunek D. (2017). Influence of various parameters on strength and absorption properties of fly ash based geopolymer concrete designed by Taguchi method. Constr. Build. Mater..

[39] Sharifi E., Sadjadi S.J., Aliha M.R.M., Moniri A. (2020). Optimization of high-strength self-consolidating concrete mix design using an improved Taguchi optimization method. Constr. Build. Mater..

[40] Taguchi G., Chowdhury S., Wu Y. (2005). Taguchi’s Quality Engineering Handbook.

[41] Herrmann A., Koenig A., Dehn F. (2018). Structural concrete based on alkali-activated binders: Terminology, reaction mechanisms, mix designs and performance. Struct. Concr..

[42] EN 12504-4 (2004). Testing Concrete-Part. 4: Determination of Ultrasonic Pulse Velocity.

[43] EN 197-1 (2011). Cement-Part. 1: Composition, Specifications and Conformity Criteria for Common Cements.

[44] Wortmann K. (2018). EP 3296278 A1, Retarder for Alkali Activated Binder.

[45] Sasaki K., Kurumisawa K., Ibayashi K. (2019). Effect of retarders on flow and strength development of alkali-activated fly ash/blast furnace slag composite. Constr. Build. Mater..

[46] Amziane S. (2006). Setting time determination of cementitious materials based on measurements of the hydraulic pressure variations. Cem. Concr. Res..

[47] Stefanou G.D., Larsinos C. (1981). Influence of mixing water on the setting time of concrete. Int. J. Cem. Compos. Lightweight Concr..

[48] Robeyst N., Gruyaert E., Grosse C.U., De Belie N. (2008). Monitoring the setting of concrete containing blast-furnace slag by measuring the ultrasonic p-wave velocity. Cem. Concr. Res..

[49] Zhang Y., Zhang W., She W., Ma L., Zhu W. (2012). Ultrasound monitoring of setting and hardening process of ultra-high performance cementitious materials. NDT E Int..

[50] Mikulić D., Sekulić D., Štirmer N., Bjegović D., Grum J. (2005). Application of Ultrasonic Methods for Early Age Concrete Characterization. Application of Contemporary Non-Destructive Testing in Engineering, Proceedings of the 8th International Conference of the Slovenian Society for Non-Destructive Testing, Portorož, Slovenia, 1–3 September 2005.

[51] Zhang S., Zhang Y., Li Z. (2018). Ultrasonic monitoring of setting and hardening of slag blended cement under different curing temperatures by using embedded piezoelectric transducers. Constr. Build. Mater..

[52] Aggelis D.G., Philippidis T.P. (2004). Ultrasonic wave dispersion and attenuation in fresh mortar. NDT E Int..

[53] Kmack R.M., Kurtis K., Jacobs L., Kim J.-Y. (2009). Assessment of Air Entrainment in Fresh Cement Paste Using Ultrasonic Nondestructive Testing. J. ASTM Int..

[54] Weng L., Sagoe-Crentsil K. (2007). Dissolution processes, hydrolysis and condensation reactions during geopolymer synthesis: Part I—Low Si/Al ratio systems. J. Mater. Sci..

[55] Zhang W., Zhang Y., Liu L., Zhang G., Liu Z. (2012). Investigation of the influence of curing temperature and silica fume content on setting and hardening process of the blended cement paste by an improved ultrasonic apparatus. Constr. Build. Mater..

[56] Wang X., Taylor P., Wang K., Lim M. (2016). Monitoring of setting time of self-consolidating concrete using ultrasonic wave propagation method and other tools. Mag. Concr. Res..

[57] Molero M., Segura I., Izquierdo M.A.G., Fuente J.V., Anaya J.J. (2009). Sand/cement ratio evaluation on mortar using neural networks and ultrasonic transmission inspection. Ultrasonics.

[58] Tekle B.H., Holschemacher K., Löber P., Heiden B. (2021). Mechanical Behavior and Frost-Resistance of Alkali-Activated Cement Concrete with Blended Binder at Ambient Curing Condition. Buildings.

[59] Rao S.K., Sravana P., Rao T.C. (2016). Experimental studies in ultrasonic pulse velocity of roller compacted concrete containing GGBS and m-sand. ARPN J. Eng. Appl. Sci..

[60] Ghosh R., Sagar S.P., Kumar A., Gupta S.K., Kumar S. (2018). Estimation of geopolymer concrete strength from ultrasonic pulse velocity (UPV) using high power pulser. J. Build. Eng..

[61] NRMCA CIP 16 (2016). Flexural Strength of Concrete: Concrete in Practice.

[62] Cosmes-López M.F., Castellanos F., Cano-Barrita P.F.d.J. (2017). Ultrasound frequency analysis for identification of aggregates and cement paste in concrete. Ultrasonics.

[63] Tarun R., Naik V., Mohan M., John S.P., Malhotra V.M., Carino N.J. (2004). The Ultrasoni Pulse Velocity Method. Handbook on Nondestructive Testing of Concrete.

